# Pathogen Characteristic of Biofilm‐Related CAUTI in the Intensive Care Unit

**DOI:** 10.1155/ijm/8369920

**Published:** 2026-07-16

**Authors:** Sayyidah Auliany Aminy, Abu Tholib Aman, Ema Damayanti, Ayu Rahayu, Akhmad Taufiq, Estelle Cateau, Christine Imbert, Titik Nuryastuti

**Affiliations:** ^1^ Department of Microbiology, Faculty of Medicine, Public Health and Nursing, Universitas Gadjah Mada, Yogyakarta, Indonesia, ugm.ac.id; ^2^ Microbiology Laboratory, Dr. H. Jusuf SK Hospital, Tarakan, Indonesia; ^3^ Research Center for Food Technology and Processing, National Research and Innovation Agency (BRIN), Indonesia, brin.go.id; ^4^ Indonesian Biofilm Research Collaboration Center (IBRCC), Yogyakarta, Indonesia; ^5^ Laboratoire Ecologie et Biologie des Interactions, Université de Poitiers, Poitiers, France, univ-poitiers.fr; ^6^ Laboratoire de Parasitologie et Mycologie, CHU de Poitiers, Poitiers, France, chu-poitiers.fr

**Keywords:** antimicrobial resistance, bacteria, biofilm, CAUTI, scanning electron microscope, yeast

## Abstract

**Objectives:**

Catheter‐associated urinary tract infection (CAUTI) in the intensive care unit (ICU) is one of the healthcare‐associated infections with increased length of stay, healthcare costs, morbidity, and mortality. CAUTI can be caused by antimicrobial‐resistant and biofilm‐forming microorganisms, resulting in recurrent and persistent infections. This study is aimed at analyzing the pathogens, antimicrobial sensitivity patterns, and biofilm‐forming capabilities of CAUTI isolates in two referral hospitals in Yogyakarta, Indonesia.

**Method:**

A cross‐sectional study was conducted on 71 patients with indwelling urinary catheters from October 2022 to March 2023. Urine specimens were collected aseptically and cultured. Microorganisms were identified and tested for antimicrobial susceptibility using Vitek 2, CHROMagar, and broth microdilution. Biofilm‐producing bacteria were identified by microtiter plate assay (MTPA), and selected urinary catheters were examined by scanning electron microscopy (SEM).

**Results:**

A total of 71 urine specimens from patients with indwelling latex‐urinary catheters resulted in 49 bacterial isolates and 28 yeasts identified. *Escherichia coli*, *Acinetobacter baumannii*, and *Candida albicans* were the most frequent pathogens. Among Enterobacterales, 62.5% were extended‐spectrum *β*‐lactamase (ESBL) producers, whereas 90% of *A. baumannii* and 20% of *Pseudomonas aeruginosa* isolates were carbapenem‐resistant. In contrast, *C. albicans* and *C. tropicalis* showed a fairly good level of sensitivity to various antifungals. Biofilm formation was observed in 91.8% of bacterial and in all *Candida* isolates. SEM examination of urinary catheter segments and tips revealed biofilm structures and microbial morphologies consistent with urine culture findings.

**Conclusion:**

*E. coli*, *A. baumannii*, and *C. albicans* were the predominant CAUTI pathogens. Most isolates exhibited biofilm‐forming ability. Although bacteria showed high antibiotic resistance, yeasts remained relatively susceptible to antifungals.

## 1. Introduction

Urinary tract infections (UTIs) account for around 20%–40% of all healthcare‐associated infections (HAIs) [1–3]. Approximately 80% of UTIs are catheter‐associated urinary tract infections (CAUTIs) and occur in consequence of the use of urinary stents or catheters. The incidence of UTIs reached 400 cases in 2018 with a prevalence rate of 9.8% [4–6]. Invasive medical devices such as urinary catheters are one of the important risk factors for the occurrence of UTIs, with 45%–79% of adult patients in the ICU using urinary catheters [7–9]. CAUTI is one of the most common HAIs with a relatively high rate of mortality and morbidity. The incidence of CAUTIs in the United States reaches 1 million cases per year, with the incidence in the ICU being three to five times higher compared with regular care units [10–13].

Microorganisms that cause CAUTI in hospital settings and are associated with biofilm formation include *Escherichia coli*, *Enterococcus* spp., *Klebsiella pneumoniae*, *Enterobacter spp*., *Candida* spp., *Staphylococcus aureus*, *Proteus mirabilis*, *Pseudomonas aeruginosa*, *Acinetobacter spp.*, and other gram‐negative bacteria. Through the formation of biofilms and undergoing morphological alterations, uropathogens can be persistent and cause recurring infections with high morbidity, high mortality, increased length of hospital stay, and cost of treatment [14–17].

Several studies showed that 47%–86% of uropathogens causing CAUTIs are multidrug‐resistant (MDR) microorganisms. Recently, the incidence of CAUTIs caused by MDR microorganisms increased significantly [11, 13]. These infections are difficult to treat due to limited antimicrobial options. Microbial resistance can occur due to mutations or horizontal gene transfer, leading to changes in antibiotic targets, increased efflux pump activity, alterations in cell membrane permeability, or the production of enzymes that can modify antibiotics [18]. A history of antibiotic usage, prolonged antibiotic use, prolonged use of urinary catheter, and irrational therapy of UTIs are some of the factors that contribute to the increasing number of MDR microorganisms as a cause of CAUTI [13, 19–23].

It has been documented that 73.4% of uropathogens have the ability to form biofilms [17]. Biofilms are sessile single species or polymicrobial communities that adhere to biotic or abiotic surfaces and are encased within a self‐produced extracellular polymeric matrix. This lifestyle represents a virulence factor for pathogens, allowing them to escape host defenses and enhance antimicrobial resistance. Biofilm causes microorganisms to be 100–1000 times more resistant to antibiotics compared with their planktonic form due to the difficulty of antibiotic penetration, the presence of persister microorganisms, environmental changes, and the exchange of resistance genes [17, 24]. The use of urinary catheters, urinary catheter materials, and the formation of biofilms on urinary catheters play important roles in the pathogenesis of CAUTIs [25, 26]. Urinary catheters are the appropriate surfaces for biofilm formation, and biofilms are also ideal places for uropathogens to evade the immune system and antibiotic agents. The materials used to make urinary catheters also matter; for example, latex urinary catheters have been shown to be 1.5 times more likely than silicone catheters to form biofilms due to latex catheters′ rough surface, which has pores that can be seen under an electron microscope [26]. Factors such as the duration of urinary catheter use, suboptimal urinary catheter care, gender, comorbidities (diabetes mellitus, immunocompromised condition), history of antibiotic use, advanced age, colonization of pathogenic microorganisms in the urine bag, catheter, and urethral area, and the virulence factors possessed by uropathogens can also increase the risk of developing CAUTI [27–30]. Thus, biofilm‐related infections pose a serious threat because they are associated with chronic, recurrent infections, with low sensitivity to treatments, and increased healthcare costs [26, 31]. CAUTIs caused by MDR microorganisms and/or those capable of forming biofilm require special attention [17, 24, 32]. Research on the profile of pathogenic microorganisms causing CAUTI, including their patterns of susceptibility to antimicrobials and biofilm formation capabilities, needs to be conducted.

This study investigates pathogenic microbes associated with single‐species and polymicrobial biofilm‐related CAUTIs, along with the main structural characteristics of biofilms on catheters retrieved from ICU patients. Furthermore, it is aimed at identifying the etiologic microbial pathogens, the factors affecting their ability to form a biofilm, and their antimicrobial resistance patterns.

## 2. Material and Methods

### 2.1. Study Design and Settings

This was a cross‐sectional study conducted at the Department of Microbiology, Faculty of Medicine, Public Health, and Nursing UGM, from October 2022 to March 2023. About 71 urine specimens from patients admitted to the ICU who developed CAUTIs and had been on urinary catheters for longer than 48 h were included. These samples were obtained from Sardjito Hospital and Academic Hospital of Gadjah Mada University, Yogyakarta, Indonesia.

### 2.2. Ethical Approval of the Study Protocols

Each study participant gave written informed consent under Protocol KE/FK/0771/EC/2022, as approved by the Ethics Committee of the Faculty of Medicine, Public Health, and Nursing University of Gadjah Mada, and UGM academic hospital. All data were analyzed anonymously.

### 2.3. Sample Collection

All catheterized patients, irrespective of gender and age more than 18 years old who met the criteria of CAUTI were involved in the study. Hospital identity number, age, gender, symptoms, duration of catheterization, and type of catheter were recorded. The type of urinary catheter was recorded for each patient; among the 71 catheterized ICU patients, 66 used latex‐based urinary catheters and five used silicone‐based urinary catheters. Urine samples were collected aseptically from catheter tubing and transported to a laboratory without any delay. Urine cultures were obtained for all patients who developed symptoms of UTI, like fever (> 38°C), suprapubic tenderness, costovertebral angle pain, or tenderness after 48 h of catheterization. In addition, this study involved the collection of multiple urine catheter tip specimens, which were cultured to reveal the presence of both monomicrobial and polymicrobial microbial growth. These specimens were subsequently analyzed for biofilm formation using scanning electron microscopy (SEM).

### 2.4. Isolation, Identification, and Antibiotic Susceptibility Pattern of Uropathogen

Microorganism were isolated from urine specimens by inoculating the specimens on to blood agar and MacConkey agar plates (Oxoid, United Kingdom), followed by aerobic incubation at 37°C for 24 h. Morphologically distinct colonies were subsequently selected and subcultures onto fresh agar plates to obtain pure cultures. The purity of each isolate was verified through macroscopic colony morphology evaluation and Gram staining. Uropathogens were identified and tested for antimicrobial susceptibility using the automated Vitek 2 Compact system (bioMérieux, France) according to the manufacturer′s instructions, CHROMagar Candida Plus (E&O Laboratories Ltd.), and antimicrobial susceptibility test using the microdilution broth method. The Vitek 2 system demonstrates high reliability for species‐level identification of common clinical isolates, with the average accuracy rates between 91% and 93% for gram‐positive bacilli and gram‐negative cocci. Vitek 2 is a robust and standardized method for species identification and antimicrobial susceptibility testing (AST) in clinical research [33–35]. Statistical analysis was performed using chi‐square and Fisher′s exact tests to assess differences in biofilm‐forming ability among microorganisms and its association with MDR. A *p* value of < 0.05 was considered statistically significant. Data visualization, including donut charts, 2D pie charts, and bar charts, was generated using an online platform for data analysis and visualization (https://www.bioinformatics.com.cn/). The Performance Standards for Antimicrobial Susceptibility Testing by Clinical and Laboratory Standard Institute (CLSI) M100 were used to determine if the uropathogens are resistant or susceptible to antimicrobials. Bacterial uropathogens were classified into non‐MDR, MDR, extensively drug‐resistant (XDR), and pan‐drug‐resistant (PDR). MDR was defined as acquired nonsusceptibility to at least one agent in three or more antimicrobial categories, XDR was defined as nonsusceptibility to at least one agent in all but two or fewer antimicrobial categories (i.e., bacterial isolates remain susceptible to only one or two categories), and PDR was defined as nonsusceptibility to all agents in all antimicrobial categories [36]. Yeast uropathogens were classified into non‐MDR, MDR, and XDR. MDR yeast was defined as an isolate exhibiting nonsusceptibility to at least one agent in two or more antifungal classes, whereas XDR yeast was defined as nonsusceptibility to at least one agent in three or more antifungal classes [37].

### 2.5. Biofilm Formation Test

A biofilm formation assay was conducted using the microtiter plate assay (MTPA) method. The bacterial isolates were cultured in Trypticase soy broth (TSB) (Oxoid), whereas yeast isolates were cultured in yeast peptone dextrose (YPD) (Oxoid) medium. Briefly, one to five colonies of bacteria or yeasts were inoculated into the respective media and incubated at 37°C for 18–24 h. To confirm microbial growth, the turbidity of the media containing the colonies was assessed postincubation. A spectrophotometer was employed to adjust the turbidity to a concentration of 10^7^–10^8^ CFU/mL. The final inoculum concentration of 10^5^ CFU/mL for each isolate was achieved by appropriately diluting the bacterial and yeast mixtures prior to placement into the microplate wells.

For each isolate, 100 *μ*L of the inoculum was added to wells containing 100 *μ*L of TSB (Oxoid) for bacteria or Roswell Park Memorial Institute (RPMI) medium or RPMI 1640 (Sigma‐Aldrich) medium for yeast. Each isolate was tested in triplicate in eight wells per isolate. After 18–24 h of incubation at 37°C, the microplate was gently washed once with phosphate‐buffered saline (PBS). Following this, 125 *μ*L of 0.1% crystal violet solution was added to each well for biofilm staining. The wells were washed three times with distilled water, and 125 *μ*L of 5% isopropanol was subsequently added to solubilize the crystal violet. The optical density (OD) at 600 nm was measured using an automated ELISA reader (Microplate reader Benchmark, Bio‐RAD). TSB and RPMI media served as negative controls throughout the assay. Biofilm formation was categorized based on the average of OD values, with the cutoff optical density (ODc) defined as the average OD of the negative control plus three times the standard deviation of the negative control OD, as outlined in Table 1 [24, 38, 39].

**Table 1:** Interpretation of biofilm formation ability of isolates based on the optical density at 600 nm.

Biofilm formation ability	Average OD (OD isolate)
Nonbiofilm producer	OD isolate ≤ ODc
Weak	ODc < OD isolate ≤ 2× ODc
Moderate	2× ODc < OD isolate ≤ 4× ODc
Strong	OD isolate > 4× ODc

### 2.6. Biofilm Examination of Latex‐Urinary Catheter Tip With SEM

In this study, SEM examinations were performed on several urinary catheter specimens obtained from CAUTI patients using latex and silicone materials. Additionally, unused urinary catheter specimens were examined as a baseline for comparison. Each specimen of the distal tip of the urinary catheters was cut into a size of 1 × 1 cm, then fixed with 3% glutaraldehyde for 30 min, and subsequently washed with PBS pH 7.4 three times. The urinary catheter pieces were then dehydrated using a graded ethanol method, specifically with 50% ethanol for 10 min, 70% ethanol for 10 min, and 96% ethanol for 20 min. The dehydrated specimens were sputter‐coated with gold (Au) using a HITACHI MC 1000 at a current of 10 mA for 30 s, ensuring a uniform conductive layer without masking the fine topographical details of the biofilm surface. SEM was conducted using a HITACHI SU3500 operating at a low accelerating voltage of 3 kV to minimize beam‐induced damage to biological structures and mitigate specimen charging. High‐resolution topographical images were acquired via a Secondary Electron (SE) detector to capture the detailed surface morphology. Observations were conducted at magnifications up to 10,000× to visualize the catheter surface and the architectural complexity of the biofilm. The system maintained a high‐vacuum environment (6 Pa) to ensure electron beam stability and prevent specimen contamination during imaging.

## 3. Results

### 3.1. Demographic Data of Patients

The study included 71 ICU patients who were using urinary catheters. The demographic data are displayed in Table 2, 38 (53.52%) of the research subjects were male, and 33 (46.48%) were female. The majority of patients (69.01%, *n* = 49) were over 50 years of age. Most of the patients (98.59%, *n* = 70) had a history of previous antibiotic use, and all patients had comorbidities. In terms of microbiological findings, 49 bacterial isolates and 28 yeast species were identified. The majority of urine cultures (91.55%, *n* = 65) grew monomicrobial pathogenic microorganisms, whereas 8.45% (*n* = 6) showed polymicrobial growth (more than one microbial pathogen) and suggested a potential coinfection with both bacterial and fungal pathogens in these cases. The mean duration of urinary catheterization in patients until the onset of symptoms indicative of CAUTI and the subsequent performance of microbiological urine culture tests were 5.04 and 12.8 days for latex‐based urinary catheters and silicone‐based urinary catheters, respectively.

**Table 2:** Demographic data of the 71 studied patients with CAUTI in ICU.

Variable		Frequency (*n*)	Percentage (%)
Hospital	RSUP Dr. Sardjito	29	40.85
	UGM Academic Hospital	42	59.15
Gender	Male	38	53.52
	Female	33	46.48
Age	18 ≤ × ≤ 50	22	30.99
	>50 years	49	69.01
Comorbidities	Yes	71	100.00
	No	0	0.00
Comorbidities type	Diabetes mellitus	23	32.39
	Immunosuppressive condition	40	56.34
	Obesity	17	23.94
	Gastrointestinal disease	17	23.94
	Kidney and urinary tract diseases	27	38.03
	Cardiovascular disease	32	45.07
	Metabolic disease	46	64.79
	Neurological disease	29	40.85
	Respiratory disease	54	76.06
	Tumor and malignancy diseases	9	12.68
History of antibiotic use	Yes	70	98.59
	No	1	1.41
Urine culture result	Monomicrobial	65	91.55
	Polymicrobial (two species)	6	8.45

### 3.2. Uropathogen Profile

The bacteria obtained in this study consisted of 49 isolates as shown in Table 3, belonging to 10 bacterial species. These bacteria species comprised 34.7% gram‐positive and 65.3% gram‐negative ones. The gram‐positive bacteria species included *Enterococcus faecium*, *Enterococcus faecalis*, *Staphylococcus haemolyticus*, and *S. aureus*. The gram‐negative bacteria species obtained included *E. coli*, *A. baumannii*, *P. aeruginosa*, *K. pneumoniae*, *Enterobacter cloacae complex*, and *Burkholderia cepacia*. The yeasts obtained consisted of 28 isolates, including the species *C. albicans*, *C. tropicalis*, *Candida krusei*, *Candida parapsilosis*, *Candida glabrata*, and *Candida* spp. Some *Candida* isolates could not be identified to the species level using CHROMagar Candida, as this method has limited ability to differentiate closely related nonalbicans species.

**Table 3:** Distribution of uropathogens causing CAUTI in ICU.

Catheter type	Bacterial species	*n*(%)	Yeast species	*n*(%)
**Latex-based urinary catheter**	*E. coli*	11 (22.4)	*C. albicans*	8 (28.6)
	*A. baumannii*	9 (18.4)	*C. tropicalis*	7 (25.0)
	*E. faecium*	8 (16.3)	*C. krusei*	5 (17.9)
	*E. faecalis*	6 (12.2)	*Candida* spp.	3 (10.7)
	*P. aeruginosa*	5 (10.2)	*C. glabrata*	1 (3.6)
	*K. pneumoniae*	2 (4.1)	*C. parapsilosis*	1 (3.6)
	*E. cloacae complex*	2 (4.1)		
	*B. cepacia*	1 (2.0)		
	*S. haemolyticus*	1 (2.0)		
	*S. aureus*	1 (2.0)		
**Silicone-based urinary catheter**	*E. coli*	1 (2.0)	*C. albicans*	1 (3.6)
	*A. baumannii*	1 (2.0)	*C. tropicalis*	1 (3.6)
	*E. faecalis*	1 (2.0)	*Candida spp*	1 (3.6)
	**Total**	**49 (100)**	**Total**	**28 (100)**

### 3.3. Antimicrobial Susceptibility Profile

In this study, a high percentage of gram‐negative bacteria exhibited resistance to antibiotic agents, which is consistent with the MIC results of bacteria that shown in Table 4. The concerned antibiotics are ampicillin, ampicillin–sulbactam, cefazolin, ceftazidime, ceftriaxone, cefepime, aztreonam, gentamicin, ciprofloxacin, nitrofurantoin, and trimethoprim–sulfamethoxazole. However, these bacteria maintained a lower MIC range to ertapenem, meropenem, amikacin, and tigecycline. Sensitivity testing of Gram‐positive bacteria revealed varying degrees of susceptibility, which are observed from MIC to penicillin, gentamicin, erythromycin, ciprofloxacin, levofloxacin, ertapenem, tetracycline, and nitrofurantoin. Notably, gram‐positive bacteria in this study remained susceptible to tigecycline, linezolid, and vancomycin (Table 5). *C. albicans* and *C. tropicalis* were the most common *Candida* species found in this study, with both species exhibiting favorable susceptibility to antifungal agents such as fluconazole, voriconazole, micafungin, caspofungin, amphotericin B, and flucytosin, as shown in Table 6.

**Table 4:** MIC value ranges (*μ*g/mL) of gram‐negative uropathogens.

	AMP	SAM	TZP	KZ	CAZ	CRO	FEP	ATM	ERT	MEM	AK	GN	CIP	TGC	F	SXT
*E. coli* (*n* = 12)	≤ 2–≥ 32	≤ 2–≥ 32	≤ 4–≥ 128	≤ 4–≥ 64	≤ 1–≥ 64	≤ 1–≥ 64	≤ 1–≥ 64	≤ 1–≥ 64	≤ 0.5	≤ 0.25	≤ 2–16	≤ 1–≥ 16	0.5–≥ 4	0.5–≥ 1	≤ 16–256	≤ 20–320
*A. baumannii* (*n* = 10)	—	16–≥ 32	≥ 128	≥ 64	≥ 64	≥ 64	≥ 64	—	**IR**	≥ 16	4–≥ 64	≥ 16	≥ 4	1–4	—	≤ 20–320
*P. aeruginosa* (*n* = 5)	—	—	8–32	≥ 64	4–16	—	≤ 1–4	16	≤ 0.25–1	≤ 0.25–1	≤ 2–16	≤ 1–8	≤ 0.25–≥ 4	**IR**	—	—
*K. pneumoniae* (*n* = 2)	**IR**	≥ 32	≥ 32–≥ 128	≥ 64	4–≥ 64	≥ 64	2–≥ 64	8–≥ 64	≤ 0.5	≤ 0.25	≤ 2–16	≤ 1–≥ 16	1–≥ 4	2	64 128	40–≥ 320
*E. cloacae complex* (*n* = 2)	**IR**	≥ 32	≥ 32–≥ 128	≥ 64	16 ≥ 64	≥ 64	1–≥ 64	≥ 64	≤ 0.5–4	≤ 0.25–0.5	4–16	≥ 16	≥ 4	≥8	64–128	40–≥ 320
*B. cepacia* (*n* = 1)	—	—	≥ 128	≥ 64	4	32	8	16	—	4	≥ 64	≥ 16	≥ 4	4	—	≤ 20

*Note:* Em dash “—” denotes susceptibility testing not performed.

Abbreviations: AMP, ampicillin; AK, amikacin; ATM, aztreonam; CAZ, ceftazidime, CIP, ciprofloxacin; ER, ertapenem; F, nitrofurantoin; FEP, cefepime; GN, gentamicin; IR, intrinsic resistance; KZ, cefazolin; MEM, meropenem; *n*, number of isolates; SAM, ampicillin–sulbactam; SXT, trimethoprim–sulfamethoxazole; TGC, tigecycline; TZP, piperacillin–tazobactam.

**Table 5:** MIC value ranges (*μ*g/mL) of gram‐positive uropathogens.

	P	OXA	GN	CIP	LEV	MOX	ER	DA	QD	LZD	VA	TE	F	RIF	SXT	AZT	CRT	TGC
*E. faecium* (8)	32–≥ 64	—	—	≥8	≥8	—	≥ 8	—	1–2	2	≤ 0.5	≥ 16	256	—	—	—	—	≤ 0.12
*E. faecalis* (7)	2–16	—	—	≤ 0.5–≥ 8	0.5–≥ 8	—	0.5–≥ 8	—	2–8	1–2	1–2	≤ 1–≥ 16	≤ 16–32	—	—	—	—	≤ 0.12
*S. haemolyticus* ∗ (1)	≥ 0.5	≥ 4	≥ 16	≥ 8	≥ 8	4	≥ 8	≥ 8	≤ 0.25	2	1	≥ 16	≤ 16	≤ 0.5	≥ 320	≥ 8	≥ 8	≤ 0.5
*S. aureus* (1)	≥ 0.5	0.5	≤ 0.5	≤ 0.5	≤ 0.12	≤ 0.25	≤ 0.25	≤ 0.25	≤ 0.25	2	1	≤ 1	≤ 16	≤ 0.5	≤ 10	≤ 0.25	≤ 0.25	≤ 0.25

*Note:* Em dash “—” denotes susceptibility testing not performed. Asterisk "∗" denotes methicillin‐resistant Staphylococcus haemolyticus, as indicated by oxacillin resistance.

Abbreviations: AZT, azithromycin; CIP, ciprofloxacin; CRT, clarithromycin; DA, clindamycin; ER, erythromycin; F, nitrofurantoin; GN, gentamicin; IR, intrinsic resistance; LEV, levofloxacin; LZD, linezolid; MOX, moxifloxacin; *n*, number of isolates; OXA, oxacillin; P, penicillin; QD, quinupristin/dalfopristin; RIF, rifampicin; SXT, trimethoprim–sulfamethoxazole; TE, tetracycline; TGC, tigecycline; VA, vancomycin.

**Table 6:** MIC value ranges (*μ*g/mL) of yeasts uropathogens.

	FLU	VOR	MIC	CAS	AMB	FCY	ITR
*C. albicans* (9)	≤ 0.5–≥ 8	≤ 0.12	≤ 0.06	≤ 0.12	0.5	≤ 0.1	0.25–≥ 0.5
*C. tropicalis* (8)	≤ 0.5–≥ 32	≤ 0.12	≤ 0.06–0.5	≤ 0.12	≤ 0.25–0.5	≤ 1	0.25–≥ 0.5
*C. krusei* (5)	**IR**	≤ 0.12	0.12–0.5	0.5	0.5	8	0.06–≥ 0.5
*Candida spp.* (4)	≤ 2–≥ 32	—	≤ 0.5	—	—	—	≥ 0.5
*C. parapsilosis* (1)	2	≤ 0.12	2	1	0.5	≤ 1	—
*C. glabrata* (1)	**IR**	≤ 0.12	≤ 0.06	0.25	0.5	≤ 1	—

Abbreviations: AMB, amphotericin B; CAS, caspofungin; FCY, flucytosine; FLU, fluconazole; IR, intrinsic resistance; ITR, itraconazole; MIC, micafungin; *n*, number of isolates; VOR, voriconazole.

This study revealed that 10 out of 16 (62.5%) Enterobacterales isolates were identified as extended‐spectrum beta‐lactamase (ESBL)–producing strains, whereas 9 out of 10 (90%) *A. baumannii* isolates were classified as carbapenem‐resistant *A. baumannii* (CRA). Additionally, one out of five (20%) *P. aeruginosa* isolates were categorized as carbapenem‐resistant *P. aeruginosa* (CRPA). Overall, 81.6% (*n* = 40) of the isolates demonstrated resistance to more than three classes of antibiotics. The predominant bacterial group was MDR, accounting for 65.3% (*n* = 32), followed by that of XDR, accounting for 16.3% (*n* = 8). No isolates meeting the PDR criteria were identified in this study.

### 3.4. Biofilm Formation Capability of Uropathogens

This study found that 91.8% of the bacterial isolates exhibited the ability to form biofilms. Among the biofilm‐producing bacteria, 42.9% were classified as strong biofilm formers, 30.6% as weak biofilm formers, and 18.3% as moderate biofilm formers as shown in Table 7. In addition, all yeasts isolated were capable of biofilm formation, with the largest number in the strong and weak biofilm‐forming categories, each comprising 39.3%, followed by the weak biofilm‐forming *Candida* isolates at 21.4% as shown in Table 8.

**Table 7:** Biofilm formation ability of uropathogen bacteria causing CAUTI in ICU.

Bacterial species	Biofilm formation (*n*)				*n*	% biofilm
	Negative	Weak	Moderate	Strong		
*E. coli*	2	6	2	2	12	83.3
*A. baumannii*	—	—	2	8	10	100
*E. faecium*	1	4	1	2	8	87.5
*E. faecalis*	1	1	2	3	7	85.7
*P. aeruginosa*	—	1	1	3	5	100
*K. pneumoniae*	—	1	—	1	2	100
*E. cloacae complex*	—	—	1	1	2	100
*B. cepacia*	—	—	—	1	1	100
*S. haemolyticus*	—	1	—	—	1	100
*S. aureus*	—	1	—	—	1	100
Total *n* (%)	**4**	**15**	**9**	**21**	**49**	
	**(8.2)**	**(30.6)**	**(18.3)**	**(42.9)**	**(100)**	

**Table 8:** Biofilm formation ability of uropathogen yeasts causing CAUTI in ICU.

Yeast species	Biofilm formation *n* (%)				*n*	% biofilm
	Negative	Weak	Moderate	Strong		
*C. albicans*	—	4	1	4	9	100
*C. tropicalis*	—	2	1	5	8	100
*C. krusei*	—	2	2	1	5	100
*C. parapsilosis*	—	—	—	1	1	100
*C. glabrata*	—	1	—	—	1	100
*Candida* spp.	—	2	2	—	4	100
Total *n* (%)	**—**	**11**	**6**	**11**	**28**	
		**(39.3)**	**(21.4)**	**(39.3)**	**(100)**	

Further analysis was conducted on the microorganisms causing CAUTI derived from patients using latex‐based urinary catheters. Based on the data, the chi‐square analysis revealed a significant difference (*p* value < 0.00001) between the neutral distribution and the distribution of bacteria and yeasts according to their ability to form biofilm. Based on the analysis conducted using chi‐square, significant differences were found between the groups of strong biofilm‐forming uropathogens and nonbiofilm‐forming uropathogens, strong biofilm‐forming bacteria and nonbiofilm‐forming bacteria, and strong biofilm‐forming yeasts and nonbiofilm‐forming yeasts with *p* values of < 0.00001, 0.00067, and 0.00091, respectively. This suggests that the bacteria and yeasts responsible for CAUTI have a greater and more significant ability to form biofilms.

In this study, we found that both nonbiofilm‐forming bacteria and biofilm‐forming bacteria were dominated by MDR group bacteria, which is consistent with the results of the statistical tests conducted. Fisher′s exact test analysis indicated no statistically significant difference in biofilm formation ability between MDR and non‐MDR bacteria (*p* value 0.5687). This indicated that microorganism capability to form biofilm and bacterial resistance are not associated.

**Figure 1:**
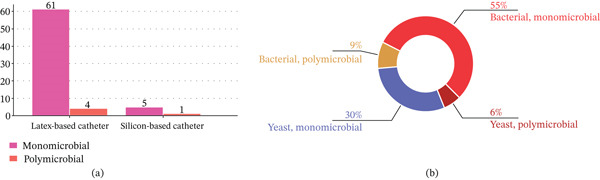
Distribution of monomicrobial and polymicrobial urine cultures. (A) Distribution according to urinary catheter material. (B) Distribution according to microorganisms.

**Figure 2:**
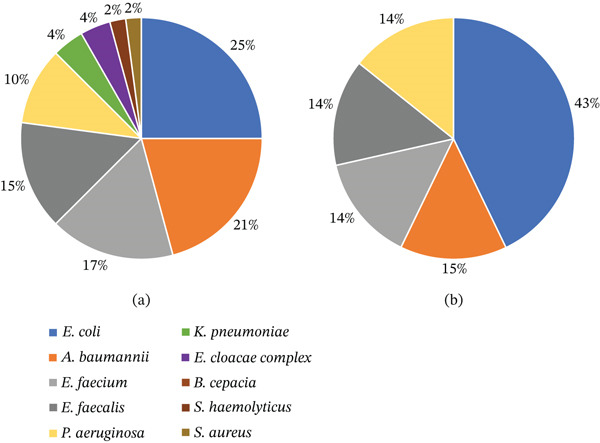
Bacteria isolated from monomicrobial and polymicrobial CAUTI cases in ICU patients. (A) Monomicrobial infections. (B) Polymicrobial infections.

**Figure 3:**
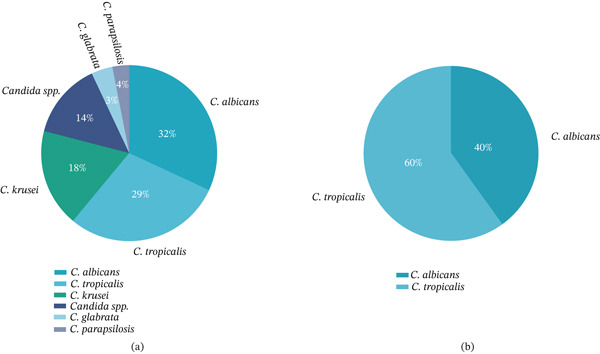
Yeasts isolated from monomicrobial and polymicrobial CAUTI cases in ICU patients. Presentation of yeasts causing (A) monomicrobial and (B) polymicrobial CAUTI in ICU patients. In this study, SEM examination was performed on unused silicone and latex urinary catheters, matching the materials used by the CAUTI patients in this research. Surface morphology analysis using SEM revealed distinct structural differences between the two types of unused catheters. The silicone urinary catheter specimen exhibited a predominantly smooth and uniform surface with an absence of significant micropores. In contrast, the latex catheter specimens demonstrated a markedly irregular topography, characterized by a rough surface and a high density of visible pores (Figure 4).

**Figure 4:**
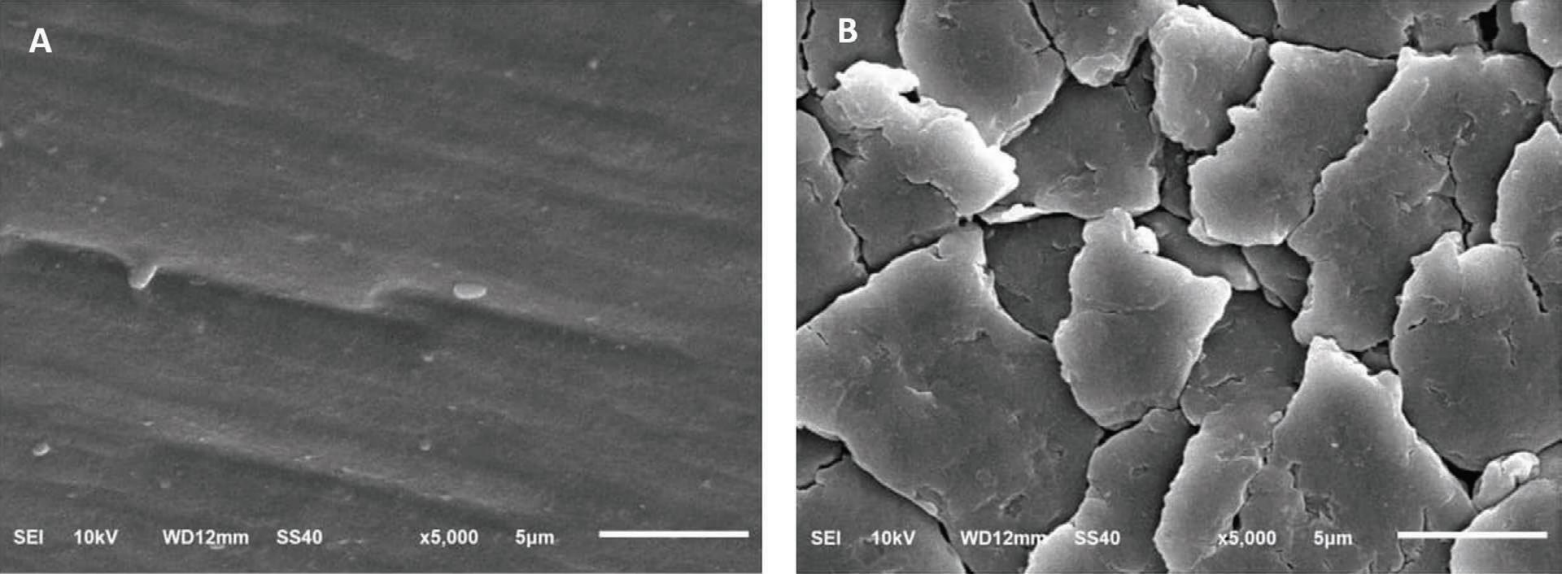
Surface structure of unused silicone and latex urinary catheters. (A) An unused silicone urinary catheter showing a smooth surface with no prominent visible pores. (B) An unused latex urinary catheter showing a rough and porous surface. Five urinary catheter tip specimens obtained from CAUTI patients were analyzed by SEM, four of which had monomicrobial and one of which had a polymicrobial urine culture. This SEM analysis revealed biofilm structures consisting of extracellular polymeric substances (EPS) surrounding uropathogens. These biofilms appeared in various forms, including honeycomb‐like, segmented, or compact structures (Figures 5, 6, and 7). Microorganisms consistent with the urine culture results were clearly identifiable in the SEM images.

This study found that 65 patients suffered from monomicrobial infections and six patients suffered from polymicrobial infections, as shown in Figure 1. In Table 9 and Figures 2, 3, and 4, as many as 83.3% (five out of six) of the polymicrobial infections were coinfections between bacteria and *Candida* spp yeast. This study analyzed the association between the microbial kingdom (categorized as fungi or bacteria) and the infection pattern (classified as monomicrobial or polymicrobial) in CAUTI. Using the chi‐square test, this study assessed whether the microbial kingdom influences the type of infection observed in patients using two different urinary catheter materials: latex‐based and silicone‐based. The statistical analysis showed no significant association between the microbial kingdom and infection pattern, with a *p* value of 0.6776, indicating that the distribution of monomicrobial versus polymicrobial infections was similar between fungi or bacteria, and irrespective of catheter material. In this study, bacterial isolates deriving from monomicrobial cultures showed varying MIC values ranging from low to high, whereas all bacterial isolates deriving from polymicrobial cultures showed high MIC values.

**Table 9:** Identification of urine culture based on the type of urinary catheter.

Type of catheter	Urine culture	Species identification
Latex‐based (*n* = 66)	Monomicrobial (*n* = 61)	*E. coli* (*n* = 9)
		*A. baumannii* (*n* = 8)
		*C. albicans* (*n* = 8)
		*E. faecium* (*n* = 7)
		*C. tropicalis* (*n* = 6)
		*E. faecalis* (*n* = 5)
		*P. aeruginosa* (*n* = 4)
		*Candida* spp. (*n* = 3)
		*C. krusei* (*n* = 2)
		*K. pneumoniae* (*n* = 2)
		*E. cloacae* (*n* = 2)
		*C. glabrata* (*n* = 1)
		*C. parapsilosis* (*n* = 1)
		*S. haemolyticus* (*n* = 1)
		*S. aureus* (*n* = 1)
		*K. pneumoniae* (*n* = 2)
		*B. cepacia* (*n* = 1)
	Polymicrobial (two species of microorganism) (*n* = 5)	*A. baumannii + C. tropicalis*
		*E. coli + C. krusei*
		*P. aeruginosa + C. Krusei*
		*E. coli + E. faecalis*
		*E. faecium + C. krusei*
Silicone‐based (*n* = 5)	Monomicrobial (*n* = 4)	*E. faecalis* (*n* = 1)
		*A. baumannii* (*n* = 1)
		*C. albicans* (*n* = 1)
		*Candida* spp.(*n* = 1)
	Polymicrobial (*n* = 1)	*E. coli* + *C. tropicalis*

**Figure 5:**
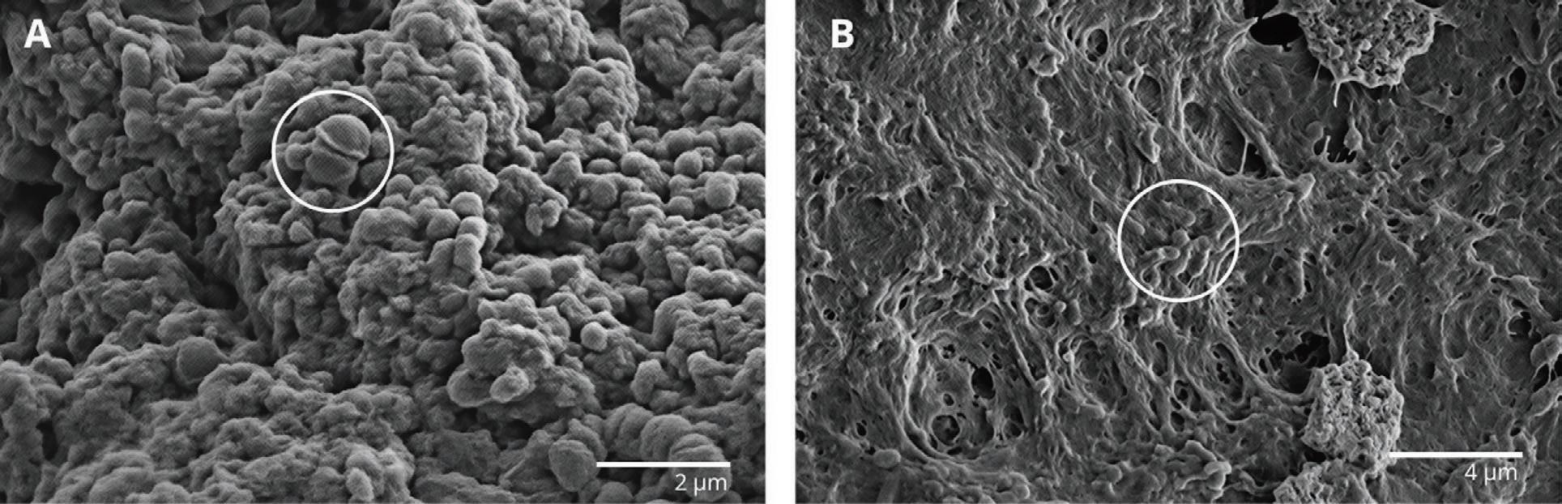
Biofilm structures on latex urinary catheter segments. (A) Specimen of a urinary catheter segment (Code 01) made of latex, used for 7 days, cultured urine revealed *E. faecium* with weak biofilm‐forming ability, showing a biofilm structure with the typical *Enterococcus* shape (oval) (white circle) surrounded by EPS. (B) Specimen of a urinary catheter segment (Code 02) made of latex, used for 8 days, urine culture revealed *E. coli* with moderate biofilm‐forming ability, showing rod‐shaped bacteria (white circle) consistent with *E. coli* surrounded by a honeycomb appearance and segmented EPS.

## 4. Discussion

This study identified uropathogenic microorganisms in CAUTI patients treated in the ICU of RSUP Dr. Sardjito and Gadjah Mada University Academic Hospital, comprising gram‐negative bacteria, gram‐positive bacteria, and yeasts. The microbial etiology of CAUTI in this study consisted of 63.4% bacteria and 36.6% *Candida* spp. The most predominant microorganisms were *E. coli* and *E. faecium*, which aligns with previous research [11, 40]. However, several studies have reported different dominant pathogens [13, 41]. The majority of yeast isolates in this study were *C. albicans* and *C. tropicalis*. Our results are consistent with the literature data that show that these two species are predominantly involved [13, 42, 43]. Variations in the microbial profile across different studies may be attributed to factors such as geographic location, hospital stay duration, patient gender, age, antibiotic usage, and local microbial patterns within the hospital [11, 44].

In this study, a high percentage of bacteria responsible for CAUTI exhibited resistance to multiple classes of antibiotics, with the antibiotic susceptibility patterns aligning with findings from previous research. The majority of *Candida* isolates, predominantly *C. albicans* and *C. tropicalis*, demonstrated good sensitivity as shown by the low MIC to voriconazole, micafungin, caspofungin, amphotericin B, and flucytosine, consistent with earlier studies (Table 6). However, a notable proportion of yeast isolates showed high MIC to itraconazole and fluconazole, with resistance rates similar to those reported in previous research [40, 45]. Specifically, 62.5% of Enterobacterales isolates were ESBL‐producing strains, 90% of *A. baumannii* isolates were carbapenem‐resistant, and 20% of *P. aeruginosa* isolates were carbapenem‐resistant. The observed ESBL prevalence was consistent with a previous report [37], but the overall percentage of ESBL‐ and carbapenem‐resistant organisms in this study is higher compared with previous reports [22, 44], possibly due to the inclusion of ICU patients with prolonged catheterization, prior antibiotic exposure, and biofilm‐associated infections. The findings of this study are consistent with prior studies, which revealed that the majority of uropathogens causing CAUTI can form biofilms and are dominated by MDR bacteria [46].

**Figure 6:**
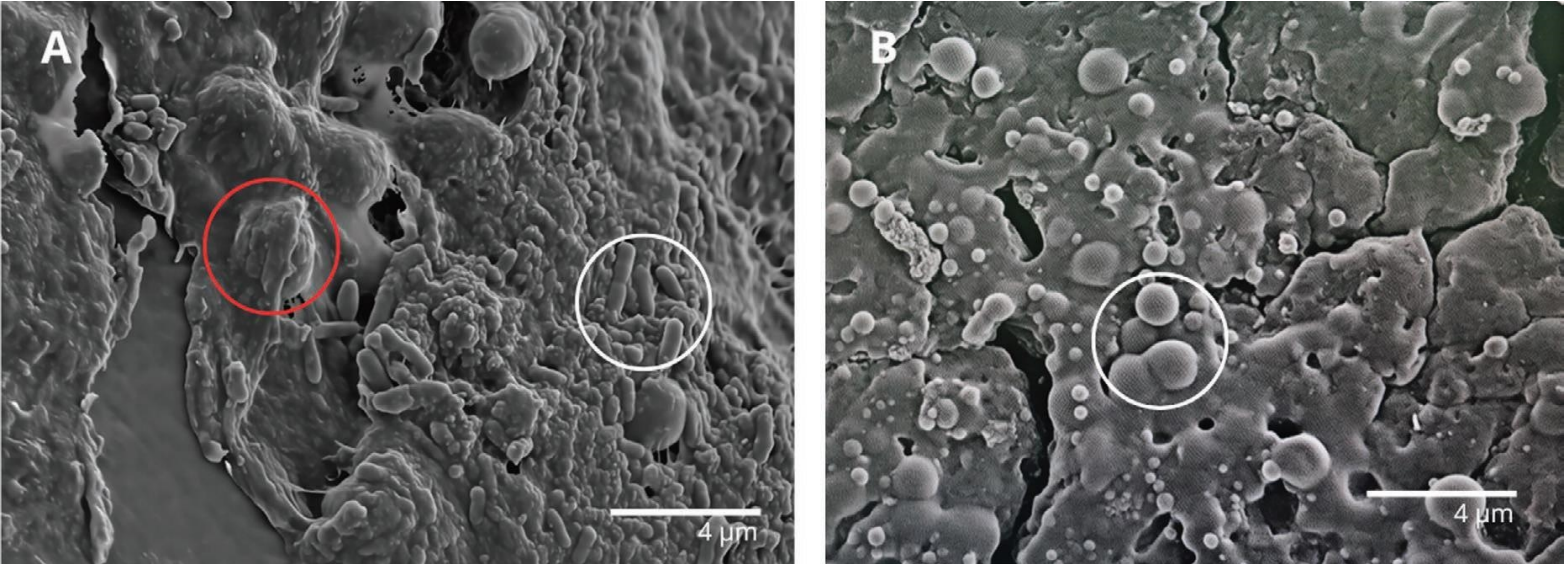
Biofilm structures on urinary catheter segments of different materials. (A) Specimen of a urinary catheter segment (Code 03) made of silicone, used for 30 days, urine cultured revealed *Candida* spp. with weak biofilm formation ability, showing yeast forms covered by fragmented and compact EPS (red circle). The visualization of the rod‐shaped bacteria that corresponds with the urine culture results in a close period (*P. aeruginosa*) (white circle). (B) A specimen of a urinary catheter segment (Code 04) made of latex, used for 8 days, urine culture showed the presence of *C. albicans* with strong biofilm‐forming ability. The visualization shows yeast forms surrounded by EPS in a solid/compact shape (white circle).

This study also found that 65.3% of the bacterial isolates were MDR, and 16.3% were potentially XDR. These findings may be attributed to factors such as irrational antibiotic use, prolonged antibiotic therapy, extended urinary catheterization, suboptimal infection prevention and control practices, and the horizontal transfer of resistance genes. These factors contribute to the rising bacterial resistance, which poses a significant threat to public health [22, 47]. The presence of antimicrobial‐resistant pathogens in CAUTI further complicates treatment, limiting therapeutic options and highlighting the need for greater attention to the management of CAUTI caused by resistant microorganisms.

The results of the biofilm‐formation ability test for microorganisms causing CAUTI revealed that 91.8% (Table 8) of bacterial isolates were capable of biofilm formation, with the majority exhibiting strong biofilm‐forming abilities (42.9%), followed by weak biofilm formers (30.6%) and moderate biofilm formers (18.3%). The results also statistically indicated that the CAUTI‐causing bacteria and yeasts have a more significant ability to form biofilms. These findings are consistent with previous studies, which have reported that 53%–85% of bacteria responsible for CAUTI are capable of biofilm formation [12, 17, 27, 45].

**Figure 7:**
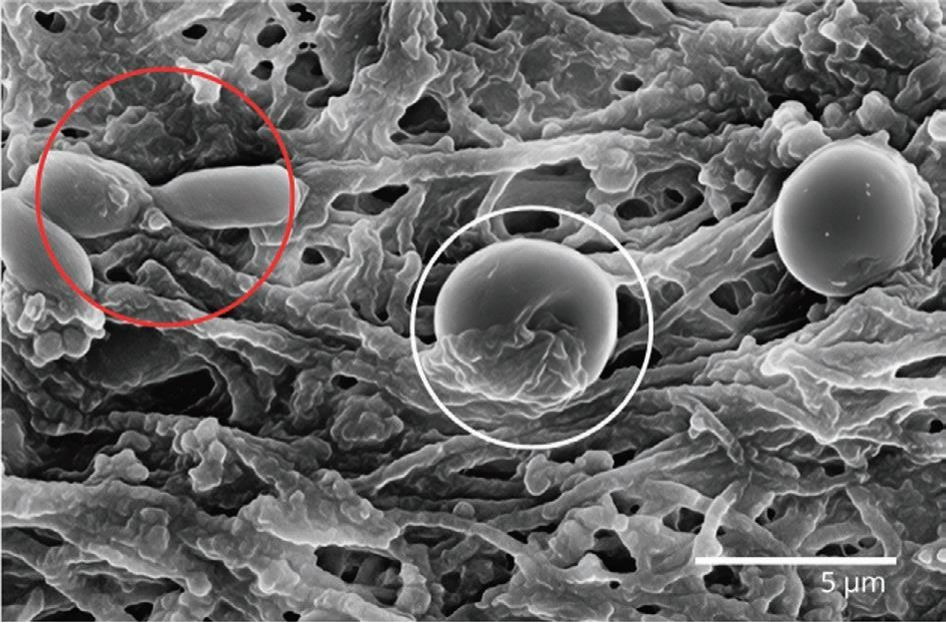
Mixed‐species biofilm on a silicone urinary catheter tip. Specimen of the urinary catheter tip made from silicone (Code 05), used for 8 days, urine culture revealed *C. albicans* with strong biofilm formation ability and *E. coli* with weak biofilm formation ability, showing yeast forms (white circles) and rod‐shaped bacteria (red circle) consistent with *E. coli* covered with EPS in a honeycomb appearance.

All *Candida* yeast isolates in this study were found to have biofilm‐forming capabilities, with 39.3% classified as strong biofilm formers, 21.4% as moderate biofilm formers, and 39.3% as weak biofilm formers. The proportion of biofilm‐forming *Candida* isolates in this study was higher than that reported in previous research, which has ranged from 30% to 65%. This discrepancy may be attributed to the relatively small number of *Candida* isolates examined in this study [48, 49].

The risk of CAUTI has been shown to increase with the duration of urinary catheter use, reaching 100% by the 28th day of catheterization [50–52]. Biofilm formation on urinary catheters is a critical factor in the pathogenesis of CAUTI. Several factors contribute to biofilm development, including trauma during catheter insertion, the coating of catheters with host proteins, and the materials used in catheter construction. Notably, latex catheters tend to support more robust biofilm formation than silicone catheters [25, 53]. The continuous flow of urine, which provides nutrients for microorganisms, along with the deposition of host proteins and glycoproteins on the catheter surface, further aids in biofilm formation [32, 54–56].

Infections caused by biofilm‐forming microorganisms are challenging to treat and often lead to persistent and recurrent infections. This difficulty arises from several factors, including limited antimicrobial penetration into the biofilm matrix, the high cellular density within the biofilm, the quorum sensing (QS) ability of microorganisms, the reduced growth rate of microorganisms in biofilms, enhanced efflux pump activity, high mutation rates, the presence of persister cells, the overexpression of resistance genes, and the exchange of resistance genes among microorganisms within the biofilm [51, 57–59].

In this study, nonbiofilm‐forming bacteria and biofilm‐forming ones were dominated by MDR bacteria. Fisher′s exact test analysis also indicated no statistically significant difference in biofilm formation ability between MDR and non‐MDR bacteria (*p* value 0.5687). These findings are consistent with prior research indicating that MDR isolates do not have a higher likelihood of biofilm production compared with non‐MDR isolates [60]. Several studies have also reported no significant correlation between antibiotic resistance and biofilm formation, and no marked differences in the expression of virulence factors, including biofilm formation, between MDR and non‐MDR bacteria [52, 60–62]. This is in line with a study by Bardoloi and Yogeesha, which indicated that microorganism capability to form biofilm and bacterial resistance are not associated [46].

Within biofilms, two key behaviors can explain the poor activity of antimicrobial agents: resistance and tolerance. Resistance refers to a process where mutations or horizontal gene transfer lead to changes in antibiotic targets, increased efflux pump activity, altered membrane permeability, or the production of enzymes that modify antibiotics. Tolerance, on the other hand, is the ability of microbial cells to endure antibiotic exposure due to reversible phenotypic changes. This includes mechanisms such as the biofilm matrix components preventing antibiotic penetration, variation in microbial colony characteristics within the biofilm and the presence of persister cells with low metabolic activity, which are more active in biofilms than in planktonic states [18, 63, 64].

In this study, biofilm structure was analyzed using SEM on four urinary catheter tip segments that showed monomicrobial growth, and one segment that showed polymicrobial growth. SEM revealed detailed surface morphology and biofilm structure, with microorganisms encased in an EPS matrix, confirming the biofilm formation corresponding to the uropathogens identified in the urine cultures [65, 66]. This is consistent with previous research, which showed that the distal part and the lumen of the urinary catheter are the most common sites for biofilm formation [32].

This study found that the use of latex‐based urinary catheters was more commonly observed compared with silicone‐based urinary catheters: 92.9% of patients used latex urinary catheters, consistent with earlier reports [13, 16]. Due to their flexibility, durability, and affordable price compared with other materials like pure silicone, latex urinary catheters are frequently utilized. However, certain people are susceptible to allergies to latex, and it also has low biocompatibility, is susceptible to bacterial infections, and has encrustations [17, 20, 67]. Compared with silicone‐based catheters, latex‐based catheters have a higher risk of biofilm formation [17, 68]. According to SEM examination, the latex catheter′s rough, porous surface was the reason for approximately 1.5 times higher biofilm growth compared with that of the silicone‐based urinary catheter. Urinary tract mucosa is known to be more irritated by latex‐based urinary catheters. As reported by Kaistha [26], silicone‐based urinary catheters are more effective than latex‐based ones at preventing the formation of biofilms. Silicone‐based urinary catheters also exhibit a lower incidence of bacterial colonization and biofilm formation. The use of latex‐based catheters is also associated with an increased risk of CAUTI [17, 20, 26, 42, 67, 68].

This study also found the dominance of monomicrobial urine culture growth results compared with polymicrobial on both materials used to form urinary catheters (Table 9). The results of this study differ from the previous research that reported polymicrobial dominance in catheter [69]. This disparity could be attributed to the use of sonication on the specimens in their research procedure. In our study, polymicrobial urine culture was dominated by coinfection between bacteria and the yeast *Candida*. This is consistent with previous research showing that bacterial UTI or bacteriuria is a risk factor for the occurrence of candiduria [70]. In this study, there was one case of coinfection of *E. coli* with *E. faecalis* in users of latex‐based urinary catheters, which is consistent with previous research that found *E. coli* enhances the growth of *E. faecalis* in artificial urine medium, where *E. faecalis* in monoculture does not grow well [71]. This study found that all bacterial isolates derived from polymicrobial cultures had high MIC values. This is consistent with previous research showing that isolates from polymicrobial cultures exhibited elevated resistance and showed multidrug as well as extensive drug resistance [72].

The study does have some limitations. First, the number of isolates examined was relatively small. Second, the AST was conducted on the planktonic phase, not the biofilm phase, which may not accurately reflect the antibiotic and antifungal resistance encountered in vivo. However, the results still suggest that 90% of uropathogens in this study were capable of forming biofilms. This has important clinical implications, as biofilm formation can make microorganisms more resistant and tolerant to antimicrobial agents, even if these agents are effective in vitro but fail to produce clinical improvement. Additionally, the urinary catheter specimens examined using SEM were not cultured, preventing a direct comparison of the SEM images with the culture results.

Despite these limitations, the findings of this study underscore the high incidence of CAUTI caused by antimicrobial‐resistant and biofilm‐forming microorganisms. Mechanisms such as phenotypic changes, gene expression alterations, reduced metabolism, and the protective EPS matrix contribute to the resistance and tolerance of microorganisms to antimicrobial therapies. As a result, comprehensive management strategies for CAUTI are essential, including the prompt replacement of urinary catheters when infection, obstruction, or system failure occurs, the rational selection of antimicrobial therapies based on microorganism sensitivity, and the implementation of CAUTI prevention bundles. These bundles include strategies such as minimizing unnecessary catheter use, employing aseptic techniques during insertion, removal, and urine specimen collection, daily review of catheter necessity, and the timely removal of unnecessary catheters. Furthermore, selecting appropriate urinary catheter materials—such as silicone, which tends to support less biofilm formation than latex—and exploring advanced nanoparticle coatings, such as antimicrobial peptides or silver nanoparticles, intermittent catheterization to shorten the catheter indwelling time, may help reduce biofilm formation on catheters [25, 32, 51, 73–75]. This study provides valuable insights into the management of biofilm‐related infections in CAUTI patients, particularly in ICU settings. The findings can inform the development of empirical antibiotic use policies, guide the hospital′s antimicrobial stewardship programs, and contribute to infection prevention and control efforts aimed at reducing CAUTI incidence and the transmission of MDR microorganisms. Additionally, these results are expected to stimulate further research into biofilm‐forming microorganisms and their role in CAUTI.

## 5. Conclusion

In conclusion, this study underscores the evolving landscape of CAUTIs within the ICU, characterized by a predominant presence of *E. coli*, *A. baumannii*, and *Candida* species. The alarming levels of MDR observed among bacterial isolates, contrasted with the relative susceptibility of fungal pathogens, highlight a divergent therapeutic challenge. Furthermore, the significant capacity for biofilm formation among these isolates further complicates treatment. These findings emphasize the urgent need for enhanced infection control measures and the development of antibiofilm strategies to improve clinical outcomes in critically ill patients.

## Author Contributions

All authors contributed to the research in various capacities, including patient selection, sample collection, microorganism identification, antimicrobial susceptibility testing, biofilm assays, data analysis, and manuscript review.

## Funding

This study was supported by Lembaga Pengelola Dana Pendidikan (10.13039/501100014538; 014/E5/PG.02.00/PRPB BATCH 2/2024).

## Ethics Statement

The study involving human participants was reviewed and approved by the Medical and Health Research Ethics Committee (MHREC) of Gadjah Mada University–Dr. Sardjito Hospital, under Approval Number KE/FK/0771/EC/2022. Written informed consent was obtained from all patients/participants involved in this research.

## Conflicts of Interest

The authors declare no conflicts of interest.

## Data Availability

The data that support the findings of this study are available on request from the corresponding author. The data are not publicly available due to privacy or ethical restrictions.

## References

[1] Chenoweth C. E. and Saint S. , Urinary Tract Infections, Infectious Disease Clinic. (2011) 25, no. 1, 103–115, 10.1016/j.idc.2010.11.005. 21315996

[2] Delost M. D. , DIAGNOSTIC for the Laboratory Sciences Introduction to, 2015, Jones & Bartlett Learning.

[3] Flores-Mireles A. , Hreha T. N. , and Hunstad D. A. , Pathophysiology, Treatment, and Prevention of Catheter-Associated Urinary Tract Infection, Topics in Spinal Cord Injury Rehabilitation. (2019) 25, no. 3, 228–240, 10.1310/sci2503-228, 31548790. 31548790 PMC6743745

[4] Zeng Z. , Zhan J. , Zhang K. , Chen H. , and Cheng S. , Global, Regional, and National Burden of Urinary Tract Infections From 1990 to 2019: An Analysis of the Global Burden of Disease Study 2019, World Journal of Urology. (2022) 40, no. 3, 755–763, 10.1007/s00345-021-03913-0, 35066637. 35066637

[5] He Y. , Zhao J. , Wang L. , Han C. , Yan R. , Zhu P. , Qian T. , Yu S. , Zhu X. , and He W. , Epidemiological Trends and Predictions of Urinary Tract Infections in the Global Burden of Disease Study 2021, Scientific Reports. (2025) 15, no. 1, 10.1038/s41598-025-89240-5. PMC11807111 39922870

[6] Alhazmi A. H. , Alameer K. M. , Abuageelah B. M. , Alharbi R. H. , Mobarki M. , Musawi S. , Haddad M. , Matabi A. , and Dhayhi N. , Epidemiology and Antimicrobial Resistance Patterns of Urinary Tract Infections: A Cross-Sectional Study from Southwestern Saudi Arabia, 2023, Medicina. 10.3390/medicina59081411 PMC10456825 37629701

[7] Nicolle L. E. , Infections Associated With Urinary Catheters, 2015, Cambridge University Press, 10.1017/CBO9781139855952.122.

[8] Dudeck M. A. , Edwards J. R. , Allen-Bridson K. , Gross C. , Malpiedi P. J. , Peterson K. D. , Pollock D. A. , Weiner L. M. , and Sievert D. M. , National Healthcare Safety Network Report, Data Summary for 2013 Device-Associated Module, American Journal of Infection Control. (2015) 43, no. 3, 206–221, 10.1016/j.ajic.2014.11.014, 25575913. 25575913 PMC4653815

[9] Saleem M. , Syed Khaja A. S. , Hossain A. , Alenazi F. , Said K. B. , Moursi S. A. , Almalaq H. A. , Mohamed H. , Rakha E. , and Mishra S. K. , Catheter-Associated Urinary Tract Infection in Intensive Care Unit Patients at a Tertiary Care Hospital, Hail, Kingdom of Saudi Arabia, Diagnostics. (2022) 12, no. 7, 10.3390/diagnostics12071695, 35885599. PMC9322978 35885599

[10] Foxman B. , The Epidemiology of Urinary Tract Infection, Nature Reviews Urology. (2010) 7, no. 12, 653–660, 10.1038/nrurol.2010.190. 21139641

[11] Peng D. , Li X. , Liu P. , Luo M. , Chen S. , Su K. , Zhang Z. , He Q. , Qiu J. , and Li Y. , Epidemiology of Pathogens and Antimicrobial Resistanceof Catheter-Associated Urinary Tract Infections in Intensivecare Units: A Systematic Review and Meta-Analysis, American Journal of Infection Control. (2018) 46, no. 12, e81–e90, 10.1016/j.ajic.2018.07.012, 30174256. 30174256

[12] Ramadan R. , Omar N. , Dawaba M. , and Moemen D. , Bacterial Biofilm Dependent Catheter Associated Urinary Tract Infections: Characterization, Antibiotic Resistance Pattern and Risk Factors, Egyptian Journal of Basic and Applied Sciences. (2021) 8, no. 1, 64–74, 10.1080/2314808X.2021.1905464.

[13] Bizuayehu H. , Bitew A. , Abdeta A. , and Ebrahim S. , Catheter-Associated Urinary Tract Infections in Adult Intensive Care Units at a Selected Tertiary Hospital, Addis Ababa, Ethiopia, PLoS One. (2022) 17, no. 3, e0265102, 10.1371/journal.pone.0265102, 35316286. 35316286 PMC8939826

[14] Flores-Mireles A. L. , Walker J. N. , Caparon M. , and Hultgren S. J. , Urinary Tract Infections: Epidemiology, Mechanisms of Infection and Treatment Options, Nature Reviews Microbiology. (2015) 13, no. 5, 269–284, 10.1038/nrmicro3432, 25853778. 25853778 PMC4457377

[15] Maharjan G. , Khadka P. , Siddhi Shilpakar G. , Chapagain G. , and Dhungana G. R. , Catheter-Associated Urinary Tract Infection and Obstinate Biofilm Producers, Canadian Journal of Infectious Diseases and Medical Microbiology. (2018) 2018, no. 1, 10.1155/2018/7624857, 7624857, 30224941. 30224941 PMC6129315

[16] Paul R. , Saha P. , Pal K. , Kapoor A. , Sinha A. , and Roy J. , Study of Biofilm Formation Among Uropathogens Isolated From Catheter-Associated UTI Patient From a Tertiary Care Hospital, Asian Journal of Medical Sciences. (2024) 15, no. 2, 126–132, 10.3126/ajms.v15i2.59265.

[17] Sabir N. , Ikram A. , Zaman G. , Satti L. , Gardezi A. , Ahmed A. , and Ahmed P. , Bacterial Biofilm-Based Catheter-Associated Urinary Tract Infections: Causative Pathogens and Antibiotic Resistance, American Journal of Infection Control. (2017) 45, no. 10, 1101–1105, 10.1016/j.ajic.2017.05.009, 28629757. 28629757

[18] Mah T. F. , Biofilm-Specific Antibiotic Resistance, Future Microbiology. (2012) 7, no. 9, 1061–1072, PMID: 22953707 10.2217/fmb.12.76. 22953707

[19] Almalki M. A. and Varghese R. , Prevalence of Catheter Associated Biofilm Producing Bacteria and Their Antibiotic Sensitivity Pattern, Journal of King Saud University-Science. (2020) 32, no. 2, 1427–1433, 10.1016/j.jksus.2019.11.037.

[20] Mohamed A. H. , Sheikh Omar N. M. , Osman M. M. , Mohamud H. A. , Eraslan A. , and Gur M. , Antimicrobial Resistance and Predisposing Factors Associated With Catheter-Associated UTI Caused by Uropathogens Exhibiting Multidrug-Resistant Patterns: A 3-Year Retrospective Study at a Tertiary Hospital in Mogadishu, Somalia, Tropical Medicine and Infectious Disease. (2022) 7, no. 3, 10.3390/tropicalmed7030042, 35324589. PMC8948891 35324589

[21] Salman N. A. M. , Mohamed M. F. , Abu Elwafa W. A. , and Goda A. M. , Isolation of Gram-Negative Organisms Causing Nosocomial Catheter Associated Urinary Tract Infection and Detection of Fosfomycin Effect on Multi-Drug Resistant Strains in Sohag University Hospital, Egyptian Journal of Medical Microbiology. (2023) 32, no. 3, 99–108, 10.21608/ejmm.2023.307754.

[22] Asmare Z. , Awoke T. , Genet C. , Admas A. , Melese A. , and Mulu W. , Incidence of Catheter-Associated Urinary Tract Infections by Gram-Negative Bacilli and Their ESBL and Carbapenemase Production in Specialized Hospitals of Bahir Dar, Northwest Ethiopia, Antimicrobial Resistance & Infection Control. (2024) 13, no. 1, 1–12, 10.1186/s13756-024-01368-7, 38273339. 38273339 PMC10809431

[23] Murugesan R. C. , Subbiah G. , Lansingh G. P. , and Santhanakumarasamy P. , Catheter - Associated Urinary Tract Infection and Biofilms: Dreaded Duo in Health Settings, Journal of Current Research in Scientific Medicine. (2024) 10, no. 1, 44–49, 10.4103/jcrsm.jcrsm.

[24] Baidya S. , Sharma S. , Mishra S. K. , Kattel H. P. , Parajuli K. , and Sherchand J. B. , Biofilm Formation by Pathogens Causing Ventilator-Associated Pneumonia at Intensive Care Units in a Tertiary Care Hospital: An Armor for Refuge, BioMed Research International. (2021) 2021, no. 1, 8817700, 10.1155/2021/8817700, 34136573. 34136573 PMC8179767

[25] Davis K. , Reduction in Catheter-Associated Urinary Tract Infections (CAUTIs) Using a Silver-Coated 100% Silicone Foley Catheter Verses a Silver-Coated Latex Foley Catheter in a Northeastern U.S. Acute Care Hospital, American Journal of Infection Control. (2005) 33, no. 5, e55–e56, 10.1016/j.ajic.2005.04.059.

[26] Kaistha S. D. , Multiple Antibiotic Resistance and Biofilm Formation of Catheter Associated Urinary Tract Infection (CAUTI) Causing Microorganisms, Journal of Bacteriology & Mycology: Open Access.(2018) 6, no. 3, 217–221, 10.15406/jbmoa.2018.06.00208.

[27] Gunardi W. D. , Karuniawati A. , Umbas R. , Bardosono S. , Lydia A. , Soebandrio A. , and Safari D. , Biofilm-Producing Bacteria and Risk Factors (Gender and Duration of Catheterization) Characterized as Catheter-Associated Biofilm Formation, International Journal of Microbiology. (2021) 2021, no. 1, 8869275, 10.1155/2021/8869275, 33688348. 33688348 PMC7920707

[28] Hooton T. M. , Nosocomial Urinary Tract Infections, 2015, Eighth Edi. Mandell, Douglas, and Bennett′s Principles and Practice of Infectious Diseases, 10.1016/B978-1-4557-4801-3.00304-0.

[29] Storme O. , Tirán Saucedo J. , Garcia-Mora A. , Dehesa-Dávila M. , and Naber K. G. , Risk Factors and Predisposing Conditions for Urinary Tract Infection, Therapeutic Advances in Urology. (2019) 11, 19–28, 10.1177/1756287218814382, 31105772. PMC6502981 31105772

[30] Asmarani D. and Sianipar O. , Hubungan Tindakan Kateterisasi Urin dengan Infeksi Saluran Kemih, 2022, Universitas Gadjah Mada.

[31] Hooton T. M. , Bradley S. F. , Cardenas D. D. , Colgan R. , Geerlings S. E. , Rice J. C. , Saint S. , Schaeffer A. J. , Tambayh P. A. , Tenke P. , and Nicolle L. E. , Diagnosis , Prevention , and Treatment of Catheter- Associated Urinary Tract Infection in Adults: 2009 International Clinical Practice Guidelines From the Infectious, Diseases Society of America. Clinical Infectious Diseases. (2010) 50, 625–663, 10.1086/650482. 20175247

[32] Werneburg G. T. , Catheter-Associated Urinary Tract Infections: Current Challenges and Future Prospects, Research and Reports in Urology. (2022) 14, 109–133, 10.2147/RRU.S273663, 35402319. 35402319 PMC8992741

[33] Guo L. , Ye L. , Zhao Q. , Ma Y. , Yang J. , and Luo Y. , Comparative Study of MALDI-TOF MS and VITEK 2 in Bacteria Identification, Journal of Thoracic Disease. (2014) 6, no. 5, https://jtd.amegroups.org/article/view/2351. 10.3978/j.issn.2072-1439.2014.02.18 PMC4015025 24822115

[34] O’Hara C. and Miller J. , Evaluation of the Vitek 2 ID-GNB Assay for Identification of Members of the Family Enterobacteriaceae and Other Nonenteric Gram-Negative Bacilli and Comparison With the Vitek GNI+ Card, Journal of Clinical Microbiology. (2003) 41, no. 5, 2096–2101, 10.1128/JCM.41.5.2096-2101.2003, 12734254. 12734254 PMC154679

[35] Ligozzi M. , Bernini C. , Bonora M. G. , De Fatima M. , Zuliani J. , and Fontana R. , Evaluation of the VITEK 2 System for Identification and Antimicrobial Susceptibility Testing of Medically Relevant Gram-Positive Cocci, Journal of Clinical Microbiology. (2002) 40, no. 5, 1681–1686, 10.1128/JCM.40.5.1681-1686.2002, 11980942. 11980942 PMC130656

[36] Magiorakos A. , Srinivasan A. , Carey R. B. , Carmeli Y. , Falagas M. E. , Giske C. G. , Harbarth S. , Hindler J. F. , Kahlmeter G. , and Olsson-Liljequist B. , Bacteria: An International Expert Proposal for Interim Standard Definitions for Acquired Resistance, 2011, Microbiology, 10.1111/j.1469-0691.2011.03570.x. 21793988

[37] Arendrup M. C. and Patterson T. F. , Multidrug-Resistant *Candida*: Epidemiology, Molecular Mechanisms, and Treatment, Journal of Infectious Diseases. (2017) 216, no. supplement 3, S445–S451, 10.1093/infdis/jix131, 28911043. 28911043

[38] Stepanović S. , Vuković D. , Hola V. , Bonaventura G. D. , Djukić S. , Ćirković I. , and Ruzicka F. , Quantification of Biofilm in Microtiter Plates: Overview of Testing Conditions and Practical Recommendations for Assessment of Biofilm Production by Staphylococci, Apmis. (2007) 115, no. 8, 891–899, 10.1111/j.1600-0463.2007.apm_630.x. 17696944

[39] O’Toole G. A. , Microtiter Dish Biofilm Formation Assay, Journal of Visualized Experiments. (2011) 30, no. 47, e2437, 10.3791/2437, 21307833. PMC3182663 21307833

[40] Karuniawati A. , Gunardi W. D. , Anggraini D. , Santosaningsih D. , Saptawati L. , Cahyarini , Puspandari N. , Endraputra P. N. , Ningsih L. , Robertus T. , Sarassari R. , Prilandari L. I. , Tiyakusuma E. , Prasetyo D. S. , and Widyatmoko L. , Pola Patogen dan Antibiogram di Indonesia Tahun 2023, 2024, PAMKI.

[41] Anggi A. , Wijaya D. W. , and Ramayani O. R. , Risk Factors for Catheter-Associated Urinary Tract Infection and Uropathogen Bacterial Profile in the Intensive Care Unit in Hospitals in Medan, Indonesia, Open Access Macedonian Journal of Medical Sciences. (2019) 7, no. 20, 3488–3492, 10.3889/oamjms.2019.684, 32002081. 32002081 PMC6980809

[42] Singhal E. , Singh R. , Bhardwaj P. , and Kumari M. , *Candida* Species in Catheter Associated Urinary Tract Infection in ICU Patients at a Tertiary Care Hospital in North India: An Observational Study, Journal of Medical and Scientific Research. (2024) 12, no. 1, 11–15, 10.17727/jmsr.2024/12-2.

[43] Deorukhkar S. C. , Saini S. , Raytekar N. A. , and Sebastian M. D. , Catheter Associated Urinary Tract Candida Infections in Intensive Care Unit Patients, Journal of Clinical Microbiology and Biochemical Technology. (2016) 2, no. 1, 15–17, 10.17352/jcmbt.000010.

[44] Kitagawa K. , Shigemura K. , Yamamichi F. , Alimsardjono L. , Rahardjo D. , Kuntaman K. , Shirakawa T. , and Fujisawa M. , International Comparison of Causative Bacteria and Antimicrobial Susceptibilities of Urinary Tract Infections Between Kobe, Japan, and Surabaya, Indonesia, Japanese Journal of Infectious Diseases. (2018) 71, no. 1, 8–13, 10.7883/yoken.JJID.2017.233, 29093320. 29093320

[45] Suryawati B. , Aman A. T. , and Nuryastuti T. , Studi Bakteri Penyebab Isk Pada Pasien Dengan Kateter Urin Di Rsud Dr, 2023, Jenis Bakteri, Kepekaan Antibiotik, Kemampuan Membentuk Biofilm Dan Identifikasi Gen Adhesin. Universitas Gadjah Mada.

[46] Bardoloi V. and Yogeesha Babu K. V. , Comparative Study of Isolates From Community-Acquired and Catheter-Associated Urinary Tract Infections With Reference to Biofilm-Producing Property, Antibiotic Sensitivity and Multi-Drug Resistance, Journal of Medical Microbiology. (2017) 66, no. 7, 927–936, 10.1099/jmm.0.000525, 28703700. 28703700

[47] Gould C. , Umscheid C. A. , Agarwal R. , Kuntz G. , Pegues D. A. , and Healthcare Infection Control Practices Advisory Committee , Guidelines for Prevention of Catheter-Associated Urinary Tract Infections 2009, 2010, Infection Control & Hospital Epidemiology. 10.1086/651091 20156062

[48] Rishpana M. S. and Kabbin J. S. , Candiduria in Catheter Associated Urinary Tract Infection With Special Reference to Biofilm Production, Journal of Clinical and Diagnostic Research. (2015) 9, no. 10, DC11–DC13, 10.7860/JCDR/2015/13910.6690, 26557518. PMC4625237 26557518

[49] Malinovská Z. , Čonková E. , and Váczi P. , Biofilm Formation in Medically Important Candida Species, Journal of Fungi. (2023) 9, no. 10, 10.3390/jof9100955, 37888211. PMC10607155 37888211

[50] Fefar D. R. , Shingala H. K. , Mehta K. D. , and Shah R. V. , Comparison of Bacterial Pathogens Causing Urinary Tract Infection Among Catheterized and Non-Catheterized Patients at a Tertiary Care Hospital, Jamnagar, Journal of Pure & Applied Microbiology. (2023) 17, no. 2, 1238–1245, 10.22207/JPAM.17.2.57.

[51] Trautner B. W. and Darouiche R. O. , Role of Biofilm in Catheter-Associated Urinary Tract Infection, American Journal of Infection Control. (2004) 32, no. 3, 177–183, 10.1016/j.ajic.2003.08.005, 15153930. 15153930 PMC2963581

[52] Mota É. C. and Oliveira A. C. , Catheter-Associated Urinary Tract Infection: Why Do Not We Control This Adverse Event?, Revista da Escola de Enfermagem da USP. (2019) 53, e03452, 10.1590/S1980-220X2018007503452, 31166534. 31166534

[53] Nacey J. N. , Tulloch A. G. , and Ferguson A. F. , Catheter-Induced Urethritis: A Comparison Between Latex and Silicone Catheters in a Prospective Clinical Trial, British Journal of Urology. (1985) 57, no. 3, 325–328, 10.1111/j.1464-410X.1985.tb06354.x, 3891005. 3891005

[54] Jacobsen S. M. , Stickler D. J. , Mobley H. L. T. , and Shirtliff M. E. , Complicated Catheter-Associated Urinary Tract Infections Due to *Escherichia coli* and *Proteus mirabilis* , Clinical Microbiology Reviews. (2008) 21, no. 1, 26–59, 10.1128/CMR.00019-07, 18202436. 18202436 PMC2223845

[55] Cortese Y. J. , Wagner V. E. , Tierney M. , Devine D. , and Fogarty A. , Review of Catheter-Associated Urinary Tract Infections and In Vitro Urinary Tract Models, Journal of Healthcare Engineering. (2018) 2018, no. 1, 2986742, 10.1155/2018/2986742, 30405898. 30405898 PMC6204192

[56] Pelling H. , Nzakizwanayo J. , Milo S. , Denham E. L. , MacFarlane W. M. , Bock L. J. , Sutton J. M. , and Jones B. V. , Bacterial Biofilm Formation on Indwelling Urethral Catheters, Letters in Applied Microbiology. (2019) 68, no. 4, 277–293, 10.1111/lam.13144. 30811615

[57] Assefa M. and Amare A. , Biofilm-Associated Multi-Drug Resistance in Hospital-Acquired Infections: A Review, Infection and Drug Resistance. (2022) 15, 5061–5068, 10.2147/IDR.S379502, 36068834. 36068834 PMC9441148

[58] Kovács R. and Majoros L. , Fungal Quorum-Sensing Molecules: A Review of Their Antifungal Effect Against *Candida* Biofilms, Journal of Fungi. (2020) 6, no. 3, 10.3390/jof6030099, 32630687. PMC7559060 32630687

[59] Mirghani R. , Saba T. , Khaliq H. , Mitchell J. , Do L. , Chambi L. , Diaz K. , Kennedy T. , Alkassab K. , Huynh T. , and Elmi M. , Biofilms: Formation, Drug Resistance and Alternatives to Conventional Approaches, Aims Microbiology. (2022) 8, no. 3, 239–277, 10.3934/microbiol.2022019, 36317001. 36317001 PMC9576500

[60] Gajdács M. , Baráth Z. , Kárpáti K. , Szabó D. , Usai D. , Zanetti S. , and Donadu M. G. , No Correlation Between Biofilm Formation, Virulence Factors, and Antibiotic Resistance in *Pseudomonas aeruginosa*: Results From a Laboratory-Based In Vitro Study, Antibiotics. (2021) 10, no. 9, 10.3390/antibiotics10091134, 34572716. PMC8471826 34572716

[61] Roberts M. E. and Stewart P. S. , Modeling Antibiotic Tolerance in Biofilms by Accounting for Nutrient Limitation, Antimicrobial Agents and Chemotherapy. (2004) 48, no. 1, 48–52, 10.1128/AAC.48.1.48-52.2004, 14693517. 14693517 PMC310152

[62] De Oliveira A. , Cataneli Pereira V. , Pinheiro L. , Moraes Riboli D. F. , Benini Martins K. , and Ribeiro de Souza da Cunha M. D. , Antimicrobial Resistance Profile of Planktonic and Biofilm Cells of *Staphylococcus aureus* and Coagulase-Negative Staphylococci, International Journal of Molecular Sciences. (2016) 17, no. 9, 10.3390/ijms17091423, 27598130. PMC5037702 27598130

[63] Ciofu O. , Moser C. , Jensen P. Ø. , and Høiby N. , Tolerance and Resistance of Microbial Biofilms, Nature Reviews Microbiology. (2022) 20, no. 10, 621–635, 10.1038/s41579-022-00682-4. 35115704

[64] Djeribi R. , Bouchloukh W. , Jouenne T. , and Menaa B. , Characterization of Bacterial Biofilms Formed on Urinary Catheters, American Journal of Infection Control. (2012) 40, no. 9, 854–859, 10.1016/j.ajic.2011.10.009, 22325732. 22325732

[65] El Abed S. , Ibnsouda S. K. , Latrache H. , and Hamadi F. , Scanning Electron Microscopy (SEM) and Environmental SEM: Suitable Tools for Study of Adhesion Stage and Biofilm Formation, 2016, Scanning Electron Microscopy.

[66] Relucenti M. , Familiari G. , Donfrancesco O. , Taurino M. , Li X. , Chen R. , Artini M. , Papa R. , and Selan L. , Microscopy Methods for Biofilm Imaging: Focus on SEM and VP-SEM Pros and Cons, Biology. (2021) 10, no. 1, 10.3390/biology10010051, 33445707. PMC7828176 33445707

[67] Obaid N. A. , Almarzoky Abuhussain S. , Mulibari K. K. , Alshanqiti F. , Malibari S. A. , Althobaiti S. S. , Alansari M. , Muneef E. , Almatrafi L. , Alqarzi A. , Alotaibi N. , Mostafa A. M. , and Hagag A. , Antimicrobial-Resistant Pathogens Related to Catheter-Associated Urinary Tract Infections in Intensive Care Units: A Multi-Center Retrospective Study in the Western Region of Saudi Arabia, Clinical Epidemiology and Global Health. (2023) 21, 101291, 10.1016/j.cegh.2023.101291.

[68] Verma A. , Differences in Bacterial Colonization and Biofilm Formation Property of Uropathogens Between the Two Most Commonly Used Indwelling Urinary Catheters, Journal of Clinical and Diagnostic Research. (2016) 10, no. 6, 10.7860/jcdr/2016/20486.7939. PMC4963701 27504341

[69] Kotaskova I. , Obrucova H. , Malisova B. , Videnska P. , Zwinsova B. , Peroutkova T. , Dvorackova M. , Kumstat P. , Trojan P. , Ruzicka F. , Hola V. , and Freiberger T. , Molecular Techniques Complement Culture-Based Assessment of Bacteria Composition in Mixed Biofilms of Urinary Tract Catheter-Related Samples, Frontiers in Microbiology. (2019) 10, 10.3389/fmicb.2019.00462, 30949137. PMC6435596 30949137

[70] Gajdács M. , Dóczi I. , Ábrók M. , Lázár A. , and Burián K. , Epidemiology of Candiduria and Candida Urinary Tract Infections in Inpatients and Outpatients: Results From a 10-Year Retrospective Survey, Central European Journal of Urology. (2019) 72, no. 2, 209–214, 10.5173/ceju.2019.1909, 31482032. 31482032 PMC6715075

[71] Nye T. M. , Zou Z. , Obernuefemann C. L. , Pinkner J. S. , Lowry E. , Kleinschmidt K. , Bergeron K. , Klim A. , Dodson K. W. , Flores-Mireles A. L. , and Walker J. N. , Microbial Co-Occurrences on Catheters From Long-Term Catheterized Patients, Nature Communications. (2024) 15, no. 1, 10.1038/s41467-023-44095-0, 38168042. PMC10762172 38168042

[72] Arooj I. , Mehmood S. , Fiaz S. , Javeed M. , Javaid A. , and Genetics M. , Antibiotic Susceptibility of Monomicrobial and Polymicrobial Cultures Isolated From Chronic Wounds, 2024, Journal of Xi’an Shiyou University.

[73] Lee K. H. , Park S. J. , Choi S. J. , Uh Y. , Park J. Y. , and Han K. H. , The Influence of Urinary Catheter Materials on Forming Biofilms of Microorganisms, Journal of Bacteriology and Virology. (2017) 47, no. 1, 32–40, 10.4167/jbv.2017.47.1.32.

[74] Alqarni M. S. , Catheter-Associated Urinary Tract Infection (CAUTI) in ICU patients, Middle East Journal of Nursing. (2021) 15, no. 1, 25–33, 10.5742/MEJN2021.93799.

[75] Rajaramon S. , Shanmugam K. , Dandela R. , and Solomon A. P. , Emerging Evidence-Based Innovative Approaches to Control Catheter-Associated Urinary Tract Infection: A Review, Frontiers in Cellular and Infection Microbiology. (2023) 13, 1134433, 10.3389/fcimb.2023.1134433, 37560318. 37560318 PMC10407108

